# RING1B-BMI1 catalyzed dynamic H2AK119ub1 modification in response to sonic hedgehog signalling during pancreatic differentiation of human embryonic stem cells

**DOI:** 10.1038/s41598-025-27698-z

**Published:** 2025-11-28

**Authors:** Niloufer P. Dumasia, Prasad S. Pethe

**Affiliations:** 1 Department of Biological Sciences, Sunandan Divatia School of Science, SVKM’s NMIMS (deemed to-be) University, Mumbai, 400056 India; 2 Symbiosis Centre for Stem Cell Research (SCSCR), Symbiosis International (Deemed University), Lavale, Pune, 412115 India; 3 Present Address: Centre of Excellence in Basic Sciences, Department of Atomic Energy, University of Mumbai, Mumbai, 400098 India

**Keywords:** BMI1, Chromatin Immunoprecipitation, Polycomb group proteins, Pancreatic progenitors, RING1B, Cancer, Cell biology, Developmental biology, Molecular biology, Stem cells

## Abstract

Human embryonic stem cells (ESCs) can differentiate into the pancreatic lineage and thus can serve as *in vitro* models for normal pancreas development. Epigenetic modulators such as RING1B and BMI1, are essential lineage regulators of the pancreatic lineage. Inhibition of the sonic hedgehog (SHH) pathway is critical for development of the pancreatic lineage. In several cancers, SHH signals regulate BMI1 protein. However, it is unclear whether SHH pathway can direct RING1B and BMI1 to the promoters of pancreas-specific genes during human pancreas development to regulate them. We performed chromatin immunoprecipitation coupled with qPCR to assess if RING1B and BMI1 occupy pancreas gene promoters and repress them by adding the repressive H2AK119ub1 mark. We compared the RING1B – BMI1 occupancy at 1000 bp upstream of the pancreas specific gene promoters across cells treated with exogenous sonic hedgehog as well as the pathway inhibitor– SANT1. We observed that the SHH pathway alters the expression of crucial pancreas genes as well as regulates RING1B and BMI1 occupancy at these gene promoters. PcG protein occupancy correlated with expression of *HNF4α* and *HHEX;* but surprisingly did not affect the mRNA expression of the master transcription factor, *PDX1*. Ou results demonstrate that during pancreatic differentiation from human embryonic stem cells, the SHH signalling pathway affects occupancy of PcG proteins – RING1B and BMI1.

**Supplementary Information:**

The online version contains supplementary material available at 10.1038/s41598-025-27698-z.

## Introduction

Human embryonic stem cells (ESCs) differentiating towards the pancreatic lineage can provide a fascinating model to study normal human pancreas development. Over the years it has been demonstrated that human ESCs can differentiate into functional pancreatic β cells capable of producing insulin^1–3^. As the stem cells differentiate they require extensive changes to chromatin to facilitate gene expression patterns, which are then faithfully maintained in the differentiated cells^4–9^. Polycomb group (PcG) proteins are histone modifiers that play a crucial role in lineage specification during mouse development are also seen in human ESC lineage specification. These PcG proteins coordinate their activity to localize to a specific gene promoter by cooperating with several proteins, some of which could be effector downstream molecules of signalling pathways^10–12^.

PcG proteins are important determinant of lineage specification from multipotent cells at the interface of the developing liver, stomach and pancreas^13^. Previously, our group had shown that the SHH signalling pathway inhibits pancreatic differentiation by promoting pancreatobiliary cell differentiation^14^. It has been reported that several signalling pathways, such as JAK-STAT, WNT signalling pathway, NOTCH signalling pathway, JNK, NF κB pathways among others alter epigenetic modulators during development^15^.

Few studies in mouse development have shown that PcG protein regulate key genes in liver and pancreas development in response to morphogen gradients existing in the developing tissue^16,17^. In order for these signalling pathways to induce long-term effect in the differentiated cell progeny, they must act via the epigenetic modulators. Sonic hedgehog signalling pathway inhibition is critical for the formation of human pancreatic cells, however it has not been demonstrated if sonic hedgehog signals regulate gene expression via epigenetic modulators at promoter sites of key pancreatic genes. We aimed to understand if SHH alters the PcG proteins RING1B and BMI1 at genes critical for pancreatic cell formation by catalyzing repressive histone H2AK119ub1 mark at their promoters. We employed ChIP qPCR to understand whether the SHH pathway can alter the presence of RING1B-BMI1 and the H2AK119ub1 mark at key pancreatic gene promoters during pancreatic differentiation.

## Materials and methods

### Human embryonic stem cells

Most cytokines, growth factors, media, and supplements were acquired from Thermo Fisher Scientific (MA, USA) unless stated otherwise. The human embryonic stem cell line, KIND1, was obtained from the National Institute for Research in Reproductive and Child Health (NIRRCH), Mumbai. KIND1 cells were maintained in a serum-free, feeder-free system: cultured on truncated-recombinant human protein Vitronectin and in complete Essential 8 media containing penicillin-streptomycin. Cells were incubated at 37 °C in a humidified atmosphere with 5% CO2 and sub-cultured every 3–4 days at a 1:4 ratio using 0.5mM EDTA (Sigma-Aldrich, St. Louis, MO, USA). As the study was performed using a human embryonic stem cell line, there were no human participants involved. The study was approved by SVKM’s NMIMS Instituional Committee for Stem Cell Research (IC SCR), approval no. NMIMS/IC-SCR/004/2016.

### Directed differentiation of human ESCs

Directed differentiation of KIND1 cells towards a pancreatic lineage was carried out as previously described^14,18^. The pancreatic differentiation protocol follows a 16-day programme as the cells transition through four stages details of which are summarized in Supplementary Table S1. Undifferentiated KIND1cells showing ~75%–80% confluency were used for differentiation and harvested at days 0, 4, 8, 12, and 16 of differentiation for protein and RNA studies. Briefly, to initiate differentiation, monolayers of human ESCs were cultured in RPMI-1640 growth media (Sigma-Aldrich) supplemented with 0.025× ITS (Sigma-Aldrich), 100 ng/ml ACTIVIN A (R&D Systems), and 25 ng/ml WNT-3A (R&D Systems) for the first day and an additional three days in RPMI-1640 containing 100 ng/ml ACTIVIN A and 2% fetal bovine serum. From day 4 (definitive endoderm, DE stage) onwards, cells were cultured in DMEM/F-12 containing 2 µM all-trans-retinoic acid (Sigma-Aldrich), 50 ng/ml NOGGIN (R&D Systems) or 100 nM LDN193189 (Sigma- Aldrich), 20 ng/ml FGF4, and 1×B-27 supplement. Between days 8 to 12, as the cells transit between stages of primitive gut tube (PG) and posterior foregut (PF), cells were cultured in DMEM/F-12 containing 2 µM all-trans-retinoic acid, 50 ng/ml NOGGIN or 100 nM LDN193189, 25 ng/ml FGF10, 1× B-27 supplement, 1× Glutamax, and 1× non-essential amino acids. In the last stage of differentiation towards pancreatic endoderm stage (PE, days 12–16), cells were cultured in DMEM/F-12 containing 2 µM all-trans-retinoic acid, 50 ng/ml NOGGIN or 100 nM LDN193189, 25 ng/ml FGF10, 1× B-27 supplement, 1× Glutamax, and 1× non-essential amino acids. Between days 4 and 12 of differentiation, sonic hedgehog signals were modulated using a small molecule antagonist or agonist of the pathway. To inhibit the pathway, 0.25 µM SANT1 (Sigma-Aldrich) was added to the culture media, and the studies were termed ‘SANT1’, while cells cultured with 25 ng/ml of recombinant human SONIC HEDGEHOG (Sigma-Aldrich) were termed ‘SHH’. Control groups were differentiated in the absence of both exogenous SANT1 and SHH but were exposed to the same quantity of vehicle.

### Chromatin immunoprecipitation coupled with quantitative PCR (ChIP-qPCR)

ChIP experiments were performed essentially as described previously^19^, with certain modifications to suit the undifferentiated human ESCs and differentiating progenitors during a modulated SHH pathway. Briefly, cells were rinsed with 1X PBS and cross-linked with 1% formaldehyde (Sigma-Aldrich) solution for 10 minutes at room temperature on a rotor. Excess formaldehyde was quenched using 0.125M glycine for 10 minutes at room temperature. Cells were washed with chilled 1X PBS and lysed in lysis buffer [50 mM HEPES-KOH, 1 mM EDTA, 140 mM NaCl, 0.1% SDS, 1% Triton X-100, 1mM PMSF, and 1X Protease Inhibitor Cocktail (Amresco, Ohio, USA). Chromatin was sheared using a probe sonicator (QSonica, Connecticut, USA) to obtain fragments in the range of 150–500 bp and clarified at 20,000 g for 10 minutes at 4 °C to pellet insoluble material. After preserving 1% of the supernatant as input samples, 30 μg of chromatin was incubated overnight at 4 °C with 5–10 μg anti-BMI1 (Cell Signaling Technology, MA, USA, 6964), anti-RING1B (Abcam, Cambridge, UK, ab101273) or anti-H2AK119Ub1 (Cell Signaling Technology, 8240) conjugated to Dynabeads Protein A (Thermo Fisher Scientific) according to the manufacturer’s instructions. A negative antibody control consisting of normal rabbit IgG (Abcam, ab172730) was included for each sample. Immunoprecipitated complexes were retrieved and washed successively in buffers containing low salts [50 mM HEPES-KOH, 1 mM EDTA, 500 mM NaCl, 0.1% SDS and 1% Triton X-100], high salts [10 mM Tris, 250 mM lithium chloride, 1 mM EDTA, 0.5% sodium deoxycholate and 0.5% NP-40] and Tris EDTA [10 mM Tris and 1 mM EDTA] before eluting target antigen in ChIP elution buffer [10 mM Tris, 1 mM EDTA and 1% SDS]. DNA was further incubated at 65°C/overnight to reverse crosslink, followed by RNase A (Sigma-Aldrich) (37°C/2 hours) and proteinase K (Sigma-Aldrich) (55°C/2 hours) treatment. For ChIP-qPCR, free DNA was purified using standard Phenol:Chloroform: Isoamyl alcohol reagent and ethanol precipitation before being subjected to quantitative PCR. 100 ng of the purified DNA was used as template for amplification on the StepOne Plus Real-Time PCR system (Applied Biosystems, CA, USA) using PowerUp SYBR Green Master Mix reagent (Applied Biosystems). Target-specific primers (see Supplementary Table S2) were designed using Primer-BLAST software (https://www.ncbi.nlm.nih.gov/tools/primer-blast/). Fold enrichment for all data was obtained from biological duplicates and qPCR was performed in three independent amplifications, and signals were normalized relative to input DNA. Data was represented for time-points day8, day12, and day16 as the SHH signals were modulated at those stages of differentiation and we could study its effect. ChIP-qPCR data represented by the control, SHH, and SANT1 groups are plotted with respect to the input DNA collected prior to immunoprecipitation with the antibodies.

### Statistical analysis

All data, unless otherwise stated, were plotted as mean value ± standard error of the mean (S.E.M) of normalized values from three independent biological experiments in at least two independent replicates. P values computed using Student’s t-test. Asterisks denote P values and represent significance between SANT1/SHH treatments at specific time points. *, p < 0.05; **, p < 0.01; ***, p < 0.001; ****, p < 0.0001; ns, non-significant with p >0.05. All graphs and statistical analyses were generated using GraphPad Prism version 8 (GraphPad Software, San Diego, CA, USA; www.graphpad.com).

## Results

### Expression of transcription factor associated with pancreatic lineage by Polycomb repressive Complex (PRC1) proteins RING1B and BMI1 in response to sonic hedgehog signalling pathway

**Fig. 1:**
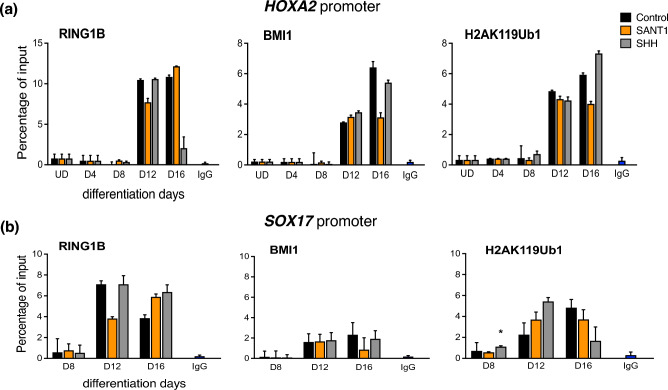
Chromatin immunoprecipitation coupled with quantitative PCR for PRC1 core subunits RING1B, BMI1, and PRC1 mark H2AK119ub1 at the promoters of pancreas-specific genes during modulation of the sonic hedgehog pathway in pancreatic progenitor formation. Occupancy studied at the promoters of *HOXA2* (**a**) and *SOX17* (**b**). Error bars denote mean ± s.e.m from three technical repeats of two independent biological studies each (n=2). P values computed using Student’s t-test. Asterisks denote P values and represent significance between SANT1/SHH treatments at specific time points. *, p < 0.05; **, p < 0.01; ***, p < 0.001; ****, p < 0.0001; ns, non-significant with p >0.05. The data in control, SHH, SANT-1 groups are compared to INPUT.

**Fig. 2:**
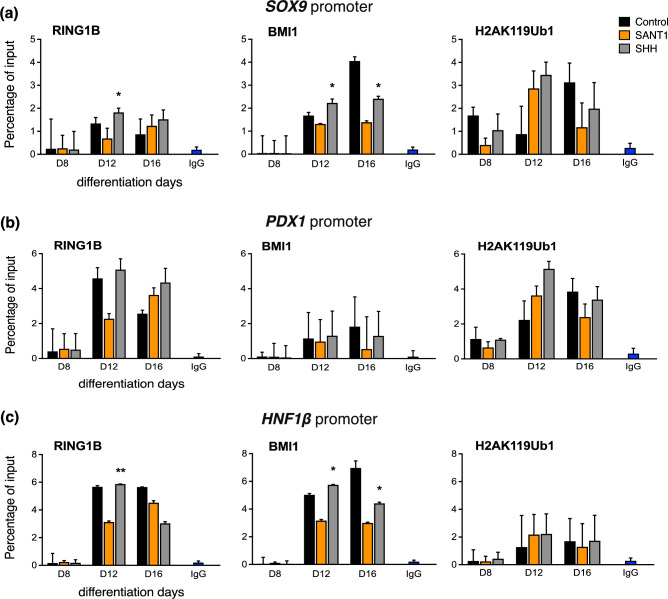
Chromatin immunoprecipitation coupled with quantitative PCR for PRC1 core subunits RING1B, BMI1, and PRC1 mark H2AK119ub1 at the promoters of pancreas-specific genes during modulation of the sonic hedgehog pathway in pancreatic progenitor formation. Occupancy studied at the promoters of *SOX9* (**a**), *PDX1* (**b**), *HNF1β* (**c**). Error bars denote mean ± s.e.m from three technical repeats of two independent biological studies each (n=2). P values computed using Student’s t-test. Asterisks denote P values and represent significance between SANT1/SHH treatments at specific time points. *, p < 0.05; **, p < 0.01; ***, p < 0.001; ****, p < 0.0001; ns, non-significant with p >0.05. The data in control, SHH, SANT1 groups are compared to INPUT.

**Fig. 3:**
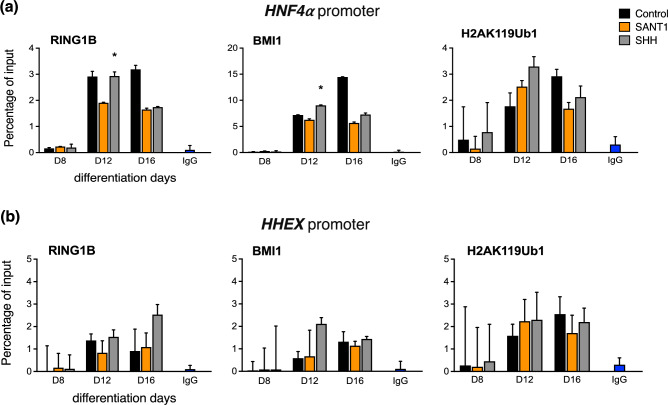
Chromatin immunoprecipitation coupled with quantitative PCR for PRC1 core subunits RING1B, BMI1, and PRC1 mark H2AK119ub1 at the promoters of pancreas-specific genes during modulation of the sonic hedgehog pathway in pancreatic progenitor formation. Occupancy studied at the promoters of *HNF4α* (**a**) and *HHEX* (**b**). Error bars denote mean ± s.e.m from three technical repeats of two independent biological studies each (n=2). P values computed using Student’s t-test. Asterisks denote P values and represent significance between SANT1/SHH treatments at specific time points. *, p < 0.05; **, p < 0.01; ***, p < 0.001; ****, p < 0.0001; ns, non-significant with p >0.05. The data in control, SHH, SANT1 groups are compared to INPUT.

The human ESCs were differentiated into pancreatic endoderm and characterized for the stage-specific transcription factors^14^. Since the SHH pathway was modulated after formation of the definitive endoderm, chromatin immunoprecipitation to analyse pancreatic gene promoters was performed between stages of primitive gut tube, posterior foregut, and pancreatic endoderm. PRC1 proteins are known to regulate *HOXA2*; hence we checked the localization of RING1B and BMI1 at 1000 bp of the *HOXA2* transcription start site. Post day 12 of differentiation, RING1B was localized to *HOXA2* promoter, as well as this also led to a high level of ubiquitylated H2A at the *HOXA2* promoter site (Figure 1a). In the differentiating cells, active SHH signals led to increased deposition of RING1B and H2AK119ub1 at the *SOX17* promoter region in the posterior foregut cells as compared to the antagonist-treated group (Figure 1b). The same was mirrored in the *SOX17* mRNA expression (data not shown, Dumasia et al., 2021). Notably, no significant change was observed in BMI1 occupancy across treatment groups in the differentiating cells at *SOX17*. Interestingly, the H2AK119ub1 mark at *SOX17* peaked at day 12, and later reduced in the SHH-treated group. This could be due to heterochromatization of the region of DNA in differentiated cells; hence, even with low H2AK119ub1, there was no expression of *SOX17*. In contrast, in the SHH antagonist group and control group, H2AK119ub1 localization remained. In case of pancreatic progenitor markers *SOX9* and *PDX1*, the localization of RING1B, BMI1 and H2AK119ub1 increased in response to active SHH signals as compared to their occupancy in SANT1-treated cells (Figure 2a & b). Remarkably, localization of PRC1 and the H2AK119ub1 mark did not correlate with the mRNA expression of *SOX9* and *PDX1*. This suggests that PRC1 does not regulate the expression of PDX1 and SOX9 during pancreatic differentiation. At the *HNF1β* promoter, activation of SHH signalling led to increased deposition of PRC1 components, BMI and RING1B, at the posterior foregut stage compared to cells treated with the pathway antagonist (Figure 2c). At the HNF4α promoter, SHH activation resulted in increased deposition of BMI1, RING1B, and H2AK119Ub1 (Figure 3a) in the posterior foregut cells (stage 3/day 12). qRT-PCR data for these genes (data not shown, Dumasia et al., 2021) indicated that SHH activation results in their transcriptional repression. A similar pattern of RING1B and BMI1 binding is observed at the *HHEX* promoter upon SHH activation; however, no parallel change in the PRC1 mark was noted (Figure 3b). Comparing PRC1 recruitment alongside the corresponding mRNA levels helped identify the potential role of PRC1 in regulating these pancreas-specific genes. Our data reveals a mechanism for SHH-mediated repression of pancreatic endoderm genes wherein active SHH signals increase BMI1 expression, which then binds developmental markers and alters cellular fate outcomes of pancreatic differentiation in humans.

### Expression of transcription factor associated with hepatic lineage by Polycomb repressive Complex (PRC1) proteins RING1B and BMI1 in response to sonic hedgehog signalling pathway

**Fig. 4:**
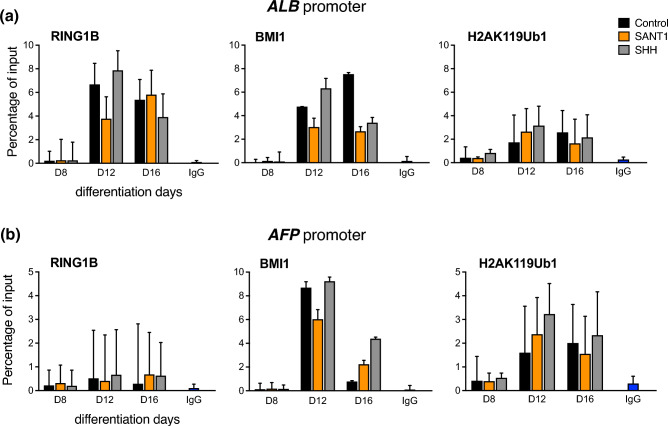
Chromatin immunoprecipitation coupled with quantitative PCR for PRC1 core subunits RING1B, BMI1, and PRC1 mark H2AK119ub1 at the promoters of liver-specific genes during modulation of the sonic hedgehog pathway in pancreatic progenitor formation. Occupancy studied at the promoters of *ALB* (**a**) and *AFP* (**b**). Error bars denote mean ± s.e.m from three technical repeats of two independent biological studies each (n=2). P values computed using Student’s t-test. Asterisks denote P values and represent significance between SANT1/SHH treatments at specific time points. *, p < 0.05; **, p < 0.01; ***, p < 0.001; ****, p < 0.0001; ns, non-significant with p >0.05. The data in control, SHH, SANT1 groups are compared to INPUT.

We also examined the promoters of liver-specific genes, since it is known that the pancreas and liver develop from a common progenitor population. The results show that occupancy of RING1B, BMI1, and H2AK119ub1 responds to the activation and inhibition of the SHH signalling pathway. RING1B does not seem to localize at the *AFP* promoter, while BMI1 seems to localize at *AFP* promoter and it correlates with the ubiquitination of H2AK119ub1. RING1B appears to be present at the *ALB* promoter, however its expression does not alter H2AK119ub1 levels across the treatment groups. At the pancreatic endoderm stage, on day 16, liver-specific promoters *ALB* and *AFP* do not display significant changes in RING1B in response to SHH modulation nor is there any significant change between the groups for the PRC1-mediated H2AK119ub1 mark. Interestingly, BMI1 seems to respond to increased SHH signals and increased localization of BMI1 is observed at liver-specific promoters as compared to RING1B occupancy and the significance of this is worth investigating (Figure 4a & b).

## Discussion

We show that SHH pathway does affect the expression of pancreas specific genes such as *SOX17* (definitive endoderm specific transcription factor)*, SOX9, PDX1, HNF4α, and HNF1β* (pancreatic endoderm specific transcription factor)*,* due to alteration in the H2AK119ub1 levels and localization of RING1B-BMI1 protein at their promoters in cells differentiating into pancreatic progenitor cells. Differential gene expression is essential during lineage specification and many histone modifiers including Polycomb group (PcG) proteins play an essential role. Our study is one of the first where we checked if PcG proteins – RING1B and BMI1 physically bind to key pancreatic gene promoters to catalyse H2AK119ub1 mark in response to sonic hedgehog signalling pathway.

Our results also show that RING1B and BMI1 are differentially localized at the promoters of key pancreas and liver specific transcription factors in response to sonic hedgehog signalling pathway during pancreatic differentiation of human ESCs. Upon transition from a pluripotent stage towards definitive endoderm repressive epigenetic marks on endoderm regulators such as SOX17, EOMES, MIXL1, and GSC are rapidly resolved by the removal of repressive PcG-mediated H3K27me3. Upon further differentiation into pancreatic endoderm the subset of DE-genes regain the H3K27me3 marks and are silenced once more^20^. We also saw that when the expression of definitive endoderm marker *SOX17* reduced there was increase in H2Ak119ub1 mark, however this was not seen in the cells that were treated with sonic hedgehog and these cells continued *SOX17* expression.

It has been demonstrated that PDX1 expression in adult human is regulated by DNA methylation and this could also be the mechanism of PDX1 regulation in pancreatic progenitor cell formation^21^. The mRNA expression of pancreatic specific transcription factor – PDX1 responded to whether SHH signalling pathway was activated or inhibited, however its expression was not affected by localization of RING1B and high level of H2Ak119ub1. PDX1 has been shown to be regulated by DNA methylation in type 2 diabetes patients^21^, and there is a possibility that same mechanism operates in normal people. Since, we observed that H2AK119ub1 mark does not affect PDX1 expression, we speculate that mechanisms such as DNA methylation might be crucial for PDX1 regulation in developing pancreatic cells. Our results show that activation of SHH signalling pathway upregulates PDX1 expression in the pancreatic progenitor cells despite deposition of the repressive H2AK119ub1 marks by RING1B, suggesting that the SHH signalling pathway may regulate DNMTs to control PDX1 expression. Recent work have showed that chromatin plays a crucial role in lineage specification into pancreatic β cells^4^, and our results also show that pancreatic gene expression regulation is complex.

We have used inhibitors of sonic hedgehog signalling pathway, which have not completely switched off this pathway and there is a possibility that downstream SHH signalling pathway components are regulated independently of the sonic hedgehog ligand. The study has limitations since we have used ChIP-qPCR for specific gene promoters only 1000 bp upstream of the transcription start site, thus we cannot determine whether histone modifications upstream of the chosen site has affected gene expression. Chromatin immunoprecipitation followed by sequencing would have yielded large data on other areas of the genome where RING1B and BMI1 bind to bring about histone modification H2AK119ub1. We cannot rule out other histone post-translational or epigenetic modifications that could dictate the gene expression during pancreatic progenitor cell formation.

Thus, our work shows that the sonic hedgehog signalling pathway affects pancreatic gene expression, and this pathway affects the localization of RING1B-BMI1 at these promoters.

## Conclusion

Our results show that the sonic hedgehog pathway can alter the occupancy of PRC1 proteins (RING1B and BMI1) at the promoters of pancreas and liver-specific genes, which also led to modified expression of the H2AK119ub1 mark at some of these promoters. However, not all the pancreas-specific genes seemed to be directly controlled by RING1B and BMI1. We demonstrate that the sonic hedgehog pathway plays an important role in modifying the localization of PRC1 proteins (RING1B and BMI1) during pancreatic differentiation of human ESCs.

## Supplementary Information

Supplementary Information 1.

Supplementary Information 2.

Supplementary Information 3.

Supplementary Information 4.

## Data Availability

The authors are willing to share data upon request. Inquiries and data requests can be directed to the corresponding author.

## Abbreviations

BMI1: B cell-specific moloney murine leukemia virus integration site 1
ChIP: chromatin immunoprecipitation
PcG: Polycomb group
PDX1: Pancreatic and duodenal homeobox 1
qPCR: quantitative polymerase chain reaction
RING1B: Ring finger protein 1B
SHH: Sonic hedgehog
SOX17: SRY (sex determining region Y)-box 17
TF: Transcription factor
