# Supplementary material for: RING1B-BMI1 catalyzed dynamic H2AK119ub1 modification in response to sonic hedgehog signalling during pancreatic differentiation of human embryonic stem cells
Source: Sci Rep. 2025 Nov 28;15:42814. doi: 10.1038/s41598-025-27698-z (PMC12663276; doi:10.1038/s41598-025-27698-z)
Supplement: Supplementary file 1 — Supplementary Information 1. [file 41598_2025_27698_MOESM1_ESM.docx]

**Supplementary Data**

RING1B-BMI1 catalyzed dynamic H2AK119ub1 modification in response to sonic hedgehog signalling during pancreatic differentiation of human embryonic stem cells

Niloufer P. Dumasia and Prasad S. Pethe

**SUPPLEMENTARY FIGURE AND TABLE LEGENDS:**

**Supplementary Figure 1:** Schematic overview of the differentiation methodology for differentiating human ESCs into pancreatic endoderm cells. (a) Summary of the four-stage protocol which includes the time points, stages of transition, important growth factors and small molecules that were added during the transitions into pancreatic endoderm cells. (b) Cells at varying stages of pancreatic differentiation were characterized using the transcription factor profile.

Abbreviations: DE - definitive endoderm, FGF2 - fibroblast growth factor 2, FGF10 - fibroblast growth factor 10, ITS - insulin-transferrin-selenium, LDN193189 hydrochloride - retinoic acid signalling pathway antagonist, PG - primitive gut tube, PF - posterior foregut, PE - pancreatic endoderm, SANT1 - sonic hedgehog antagonist, SHH - sonic hedgehog, NEAA - non-essential amino acids
