# Supplementary figures and images for: RING1B-BMI1 catalyzed dynamic H2AK119ub1 modification in response to sonic hedgehog signalling during pancreatic differentiation of human embryonic stem cells
Source: Sci Rep. 2025 Nov 28;15:42814. doi: 10.1038/s41598-025-27698-z (PMC12663276; doi:10.1038/s41598-025-27698-z)

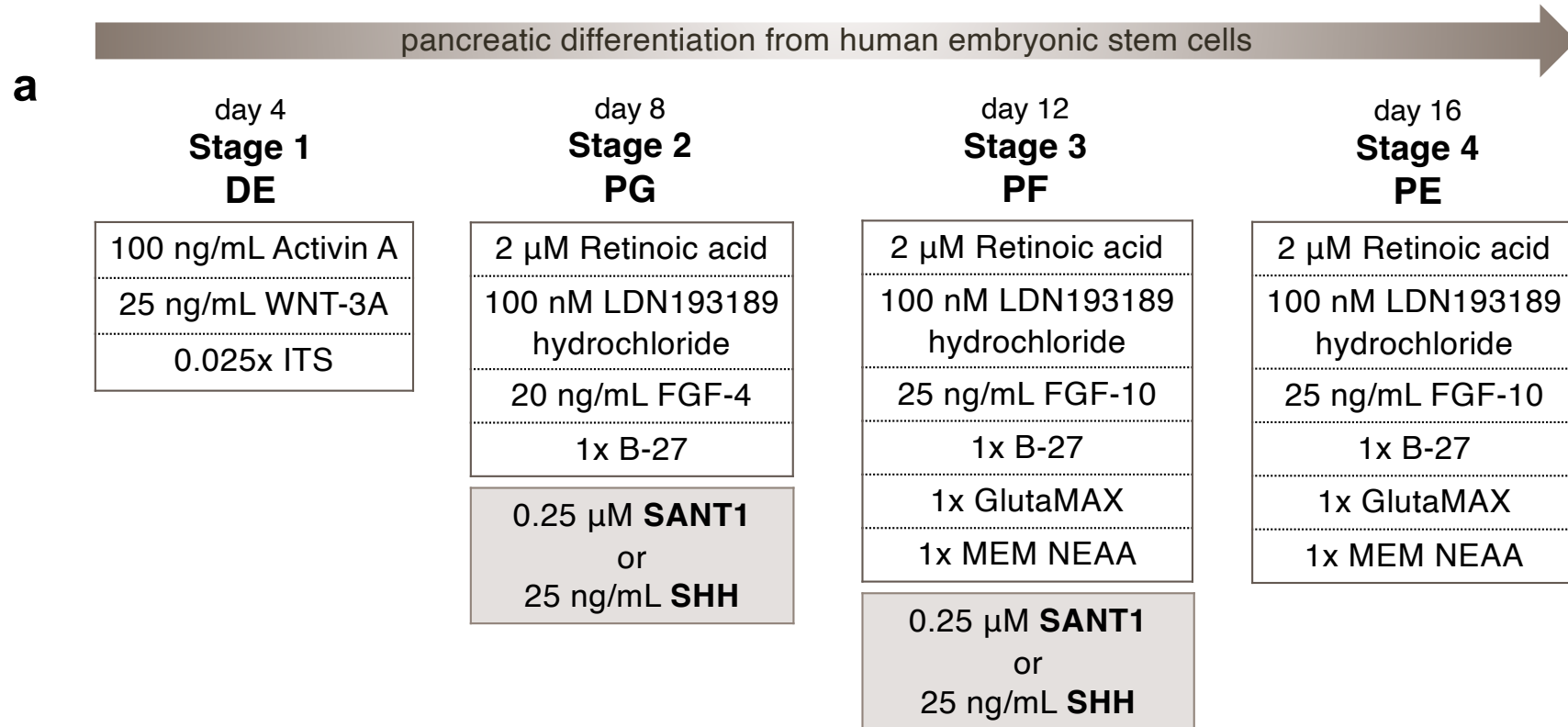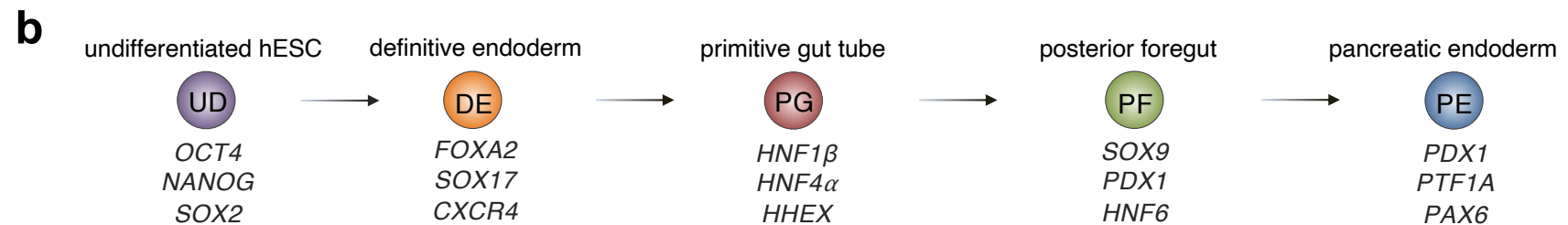

Supplement: Supplementary file 2 — Supplementary Information 2. [file 41598_2025_27698_MOESM2_ESM.pdf]
