# Supplementary material for: RING1B-BMI1 catalyzed dynamic H2AK119ub1 modification in response to sonic hedgehog signalling during pancreatic differentiation of human embryonic stem cells
Source: Sci Rep. 2025 Nov 28;15:42814. doi: 10.1038/s41598-025-27698-z (PMC12663276; doi:10.1038/s41598-025-27698-z)
Supplement: Supplementary file 3 — Supplementary Information 3. [file 41598_2025_27698_MOESM3_ESM.pdf]

Supplementary Table S1:

Details of differentiation protocol with reagents used for directed differentiation towards pancreatic lineage

Stage	Duration	Basal media	Cytokines/concentrations	Make & Catalogue number
**Stage 1**<br>**Undiff→DE**	Day 1	RPMI-1640 Sigma (R6504)	100 ng/mL ACTIVIN A	R&D Systems (338-AC/CF)
			25 ng/mL WNT-3A	R&D Systems (5036-WN/CF)
			0.025x Insulin-Transferrin-Selenium (ITS)	Sigma (I3146)
	Day 2	RPMI-1640 Sigma (R6504)	100 ng/mL ACTIVIN A	R&D Systems (338-AC/CF)
			0.2% FBS	Gibco (10270106)
	Days 3-4	RPMI-1640 Sigma (R6504)	100 ng/mL ACTIVIN A	R&D Systems (338-AC/CF)
			2% FBS	Gibco (10270106)
**Stage 2**<br>**DE→PG**	Days 1-4	DMEM Gibco (11995065)	2 μM Retinoic acid	Sigma (R2625)
			50 ng/mL NOGGIN<br>or<br>100 nM LDN193189 hydrochloride	Gibco (PHC1506)<br><br>Sigma (SML0559)
			20 ng/mL FGF-4	Gibco (PHG0154)
			1x B-27	Gibco (12587-010)
			0.25 μM SANT1<br>or<br>25 ng/mL SHH	Sigma (S4572)<br><br>Sigma (SRP3156)
**Stage 3**<br>**PG→PF**	Days 1-4	DMEM Gibco (11995065)	2 μM Retinoic acid	Sigma (R2625)
			50 ng/mL NOGGIN<br>or<br>100 nM LDN193189 hydrochloride	Gibco (PHC1506)<br><br>Sigma (SML0559)
			25 ng/mL FGF-10	Gibco (PHG0204)
			1x B-27	Gibco (12587-010)
			1x GlutaMAX	Gibco (35050-061)
			1x MEM NEAA	Gibco (11140-050)
			0.25 μM SANT1<br>or<br>25 ng/mL SHH	Sigma (S4572)<br><br>Sigma (SRP3156)
**Stage 4**<br>**PF→PE**	Days 1-4	DMEM Gibco (11995065)	2 μM Retinoic acid	Sigma (R2625)
			50 ng/mL NOGGIN<br>or<br>100 nM LDN193189 hydrochloride	Gibco (PHC1506)<br><br>Sigma (SML0559)
			25 ng/mL FGF-10	Gibco (PHG0204)
			1x B-27	Gibco (12587-010)
			1x GlutaMAX	Gibco (35050-061)
			1x MEM NEAA	Gibco (11140-050)
