# Supplementary material for: RING1B-BMI1 catalyzed dynamic H2AK119ub1 modification in response to sonic hedgehog signalling during pancreatic differentiation of human embryonic stem cells
Source: Sci Rep. 2025 Nov 28;15:42814. doi: 10.1038/s41598-025-27698-z (PMC12663276; doi:10.1038/s41598-025-27698-z)
Supplement: Supplementary file 4 — Supplementary Information 4. [file 41598_2025_27698_MOESM4_ESM.pdf]

Supplementary Table S2: List of the primers used for ChIP-qPCR from the immunoprecipitated chromatin of cells differentiating into pancreatic lineage. Primers were designed for regions 1000 bps upstream of the known transcription start site (TSS).

Gene symbol	Primer sequence (5' to 3')	Accession ID
AFP	F - TGAGCCACTCTTAGCATCCATC<br>R - CACAAGCCCTAATAAACCAAGTCC	NC_000004.12
ALB	F - GGTGAAGGTCAAGGGTTCTCAT<br>R - TGGCCCACATGTGATTCAAGC	NC_000004.12
HHEX	F - CTGAACTCCAGCCATCCGAA<br>R - TCCCTGGATTAACAGTGGCG	NC_000010.11
HNF1β	F - TCACATGTCACCCACCAAGAA<br>R - CTCCACAAGTCGCTCAGAGG	NC_000017.11
HNF4α	F - AACCCCAGAGTGCAGGACTA<br>R - CCAGTCACTTAGGGAACCCG	NC_000020.11
PDX1	F - CCCGTAGAGAGTCGTCAAGG<br>R - GCTTCCCAATACAGCGAGGG	NC_000013.11
SOX9	F - AACGTCAGAGCAGTAGCCCT<br>R - ATTAACATGCTCGGGTTCGC	NC_000017.11
SOX17	F - TCAACGGTGTCTTTCGCATT<br>R - TCTTTTTGCAGAAGCCGAGG	NC_000008.11
HOXA2	F- AGGAAAGATTTTGGTTGGGAAG<br>R- AAAAAGAGGGAAAGGGACAGAC	NC_000007.14
